# Geographic and Sociodemographic Disparities in Drive Times to Joint Commission–Certified Primary Stroke Centers in North Carolina, South Carolina, and Georgia

**Published:** 2011-06-15

**Authors:** Jenna A. Khan, Michele Casper, Mary George, G. Ishmael Williams, Linda Schieb, Sophia Greer, Andrew W. Asimos, Lydia Clarkson, Laura J. Fehrs, Dianne Enright, Khosrow Heidari, Sara L. Huston, Laurie H. Mettam

**Affiliations:** University of Illinois at Chicago School of Public Health, Chicago, Illinois. At the time of the study, Ms Khan was affiliated with the Centers for Disease Control and Prevention, Atlanta, Georgia; Centers for Disease Control and Prevention; Centers for Disease Control and Prevention, Atlanta, Georgia; Centers for Disease Control and Prevention, Atlanta, Georgia; Centers for Disease Control and Prevention, Atlanta, Georgia; Centers for Disease Control and Prevention, Atlanta, Georgia; Carolinas Medical Center, Charlotte, North Carolina; Georgia Department of Community Health, Atlanta, Georgia; Georgia Department of Community Health, Atlanta, Georgia; North Carolina State Center for Health Statistics, Raleigh, North Carolina; South Carolina Department of Health and Environmental Control, Columbia, South Carolina; Maine Center for Disease Control and Prevention, Augusta, Maine, and University of Southern Maine, Portland, Maine. At the time of the study, Dr Huston was affiliated with the University of North Carolina Gillings School of Global Public Health, Chapel Hill, North Carolina, and Tri-State Stroke Network, Raleigh, North Carolina; Tri-State Stroke Network, Raleigh, North Carolina

## Abstract

**Introduction:**

Timely access to facilities that provide acute stroke care is necessary to reduce disabilities and death from stroke. We examined geographic and sociodemographic disparities in drive times to Joint Commission–certified primary stroke centers (JCPSCs) and other hospitals with stroke care quality improvement initiatives in North Carolina, South Carolina, and Georgia.

**Methods:**

We defined boundaries for 30- and 60-minute drive-time areas to JCPSCs and other hospitals  by  using geographic information systems (GIS) mapping technology and calculated the proportions of the population living in these drive-time areas by sociodemographic characteristics. Age-adjusted county-level stroke death rates were overlaid onto the drive-time areas.

**Results:**

Approximately 55% of the population lived within a 30-minute drive time to a JCPSC; 77% lived within a 60-minute drive time. Disparities in percentage of the population within 30-minute drive times were found by race/ethnicity, education, income, and urban/rural status; the disparity was largest between urban areas (70% lived within 30-minute drive time) and rural areas (26%). The rural coastal plains had the largest concentration of counties with high stroke death rates and the fewest JCPSCs.

**Conclusion:**

Many areas in this tri-state region lack timely access to JCPSCs. Alternative strategies are needed to expand provision of quality acute stroke care in this region. GIS modeling is valuable for examining and strategically planning the distribution of hospitals providing acute stroke care.

## Introduction

Timely acute stroke care is necessary to substantially reduce disabilities that affect stroke patients (1,2). For ischemic stroke, the Food and Drug Administration has approved the use of intravenous tissue plasminogen activator (tPA) within 3 hours of symptom onset. One study suggests that the window of treatment benefit can be as long as 4.5 hours (3). However, many studies have shown that the sooner ischemic stroke patients receive tPA the greater the benefit, particularly if the treatment is initiated within 90 minutes of symptom onset, and, conversely, that delay in tPA delivery or failure to deliver tPA results in significantly worse ischemic stroke outcomes (1-5). For hemorrhagic stroke, therapeutic strategies to attenuate early hemorrhage expansion, such as early treatment for anticoagulation reversal in warfarin-related hemorrhagic strokes, are also time-sensitive (6).

Expeditious transport to hospitals capable of administering quality stroke care is an essential component of timely acute stroke care. Given that long delays can occur between onset of stroke symptoms and arrival at an emergency department (7-9) and that emergency department procedures for stroke patients often take longer than the 1 hour maximum set by the relevant National Quality Forum performance measure (8,10), drive times should be as short as possible. At least 1 state (Maryland) has already incorporated a 30-minute drive-time threshold into the destination bypass protocol for emergency medical services for patients with a suspected stroke (11).

*Recommendations for the Establishment of Stroke Systems of Care* (12), published by the American Stroke Association Task Force on the Development of Stroke Systems, recommends that "all patients having signs or symptoms of stroke be transported to the nearest primary stroke center or hospital with an equivalent designation." Joint Commission primary stroke center (JCPSC) certification is the leading nationally recognized primary stroke center designation program. Established in 2003 in response to recommendations from the Brain Attack Coalition and the American Stroke Association, JCPSC certification requires that hospitals demonstrate compliance with stroke care standards, including standardized methods for delivering clinical care based on appropriate clinical guidelines or evidence-based practice and a commitment to performance measurement and improvement of care (13). However, JCPSCs are not evenly distributed geographically; market forces and degree of local interest are among the main determinants of whether hospitals seek JCPSC certification. Reliance on JCPSCs as the sole destination facilities for acute stroke care presents challenges for developing robust stroke systems of care. Therefore, we wished to examine scenarios that could expand the number of hospitals recognized as capable of providing quality acute stroke care. The Paul Coverdell National Acute Stroke Registry (PCNASR, funded by the Centers for Disease Control and Prevention) (14) and the Get With the Guidelines–Stroke program (GWTG–Stroke, managed by the American Stroke Association) (15) are the 2 nationally recognized stroke care quality improvement initiatives. Hospitals participating in these initiatives may already have all or many of the resources needed to provide quality acute stroke care and therefore may be well positioned to be officially designated as stroke care centers with minimal additional resources.

The primary objective of this study was to document the geographic disparities in timely access to JCPSCs in the tri-state region of North Carolina, South Carolina, and Georgia, a region with a historically high burden of stroke. In addition, we examined the disparities in population access to JCPSCs by sociodemographic group. Finally, we explored the extent to which timely access to acute stroke care would improve if hospitals currently participating in nationally recognized stroke care quality initiatives (PCNASR and GWTG–Stroke) were able to meet criteria for designation as a primary stroke center.

## Methods

We conducted a drive-time analysis to determine proportions of the population living within 30- and 60-minute drive times to primary stroke centers and to hospitals participating in stroke care quality improvement programs.

We obtained 2000 population data by census tract for the tri-state region from the US Census (16). The study population for this region totaled 20,247,778 (North Carolina: 8,049,313; South Carolina: 4,012,012; and Georgia: 8,186,453). We included in our analysis all hospitals in the region, along with hospitals just across the state boundaries, that were certified as JCPSCs as of September 27, 2010 (n = 88). We also identified all hospitals that were not certified as JCPSCs but that participated in either PCNASR or GWTG–Stroke (n = 72). Participation in these programs indicates a hospital's commitment to improve the quality of stroke care. We classified and analyzed PCNASR and GWTG–Stroke hospitals that were also certified as JCPSCs only. No PCNASR hospitals were in South Carolina.

We used Network Analyst 9.3 (Environmental Systems Research Institute [ESRI], Redlands, California) and StreetMap Pro 2007 road network software (ESRI) to create drive-time areas of 30 and 60 minutes to JCPSCs. We used the geographic information system (GIS) method of aerial interpolation to estimate the population residing within 30- and 60-minute drive-time areas. We first calculated the proportion of each census tract in 30- and 60-minute drive-time areas. Next, we used that proportion to calculate the percentage of the population residing within 30- and 60-minute drive-time areas by sociodemographic characteristics. We performed χ^2^ analyses using SAS version 9.1 (SAS Institute, Inc, Cary, North Carolina).

We obtained sociodemographic data from the 2000 US Census (17). We based categories of race (black or white) and ethnicity (Hispanic or non-Hispanic) on self-reported census data. We defined urban (urbanized areas or urban clusters), rural (all nonurban areas), and poverty (income below the poverty level in 1999) on the basis of census definitions.

We overlaid drive time areas onto county-level, age-adjusted stroke death rates aggregated for the years 2001 through 2005. We defined stroke deaths as those for which the underlying cause of death on the death certificate was coded as I60-I64, I67, or I69 from the *International Statistical Classification of Diseases, 10th Revision* (18). We calculated stroke death rates per 100,000, directly age-adjusted to the 2000 US standard population. We categorized the county-level stroke death rates into quartiles and mapped them as 3 categories: bottom quartile, 2 middle quartiles, and top quartile. We did not show stroke death rates for counties with fewer than 5 stroke deaths because these rates do not meet statistical standards of reliability (19).

## Results

In the tri-state region, approximately 55% of the population resided within a 30-minute drive time to a JCPSC, and 77% resided within a 60-minute drive time to a JCPSC (Table). Stratification by age group showed a slight inverse association (ie, population percentages for both drive times decreased monotonically with increasing age group). We found variations in the proportion of the population living within a 30-minute or 60-minute drive time by race/ethnicity, education level, poverty status, and urban/rural status. We found that higher proportions of Hispanics (vs non-Hispanic blacks and non-Hispanic whites), people with more than a high school education (vs those with a high school education or less), people living in urban areas (vs those in rural areas), and people at or above the poverty level (vs those living in poverty) lived within either a 30-minute or 60-minute drive time to a JCPSC. Twenty-six percent of people living in rural areas lived within a 30-minute drive time to a JCPSC compared with 70% of those living in urban areas.

**Figure 1:**
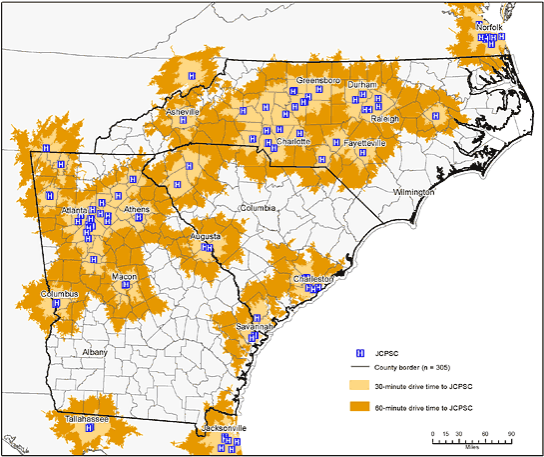
Thirty-minute and 60-minute drive time areas to a Joint Commission–Certified Primary Stroke Center (JCPSC), North Carolina, South Carolina, and Georgia.

**Figure 2:**
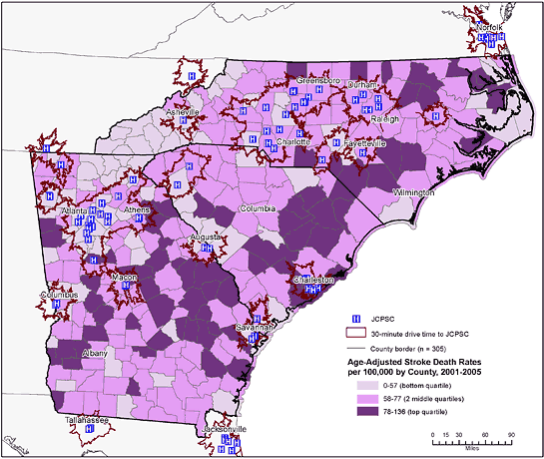
Age-adjusted stroke death rates by county and 30-minute drive-time areas to a Joint Commission–Certified Primary Stroke Center (JCPSC), North Carolina, South Carolina, and Georgia.

**Figure 3:**
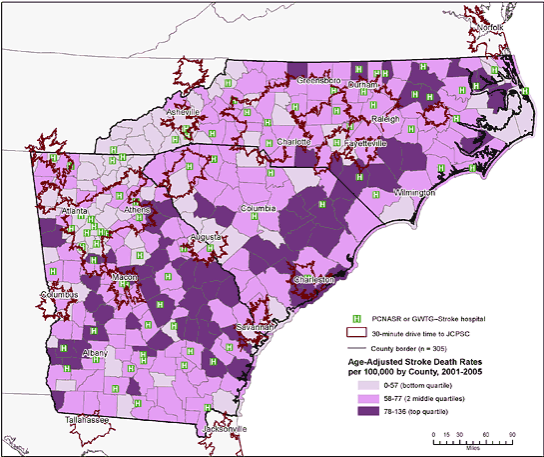
Age-adjusted stroke death rates by county, 30-minute drive-time areas to a Joint Commission–Certified Primary Stroke Center (JCPSC), and location of hospitals that participate in the Paul Coverdell National Acute Stroke Registry (PCNASR) or the Get With the Guidelines–Stroke (GWTG–Stroke) program, North Carolina, South Carolina, and Georgia.

Most JCPSCs in the region were in the northwest section along the Interstate 85 corridor (Figure 1). Few JCPSCs were in the rural, coastal plains. Stroke death rates also varied geographically in the region. Counties in the top quartile for stroke deaths (78-136 stroke deaths/100,000) were primarily in the rural coastal plains, and counties in the lowest quartile (<58 stroke deaths/100,000) were found predominantly in the piedmont and mountain areas. Many of the counties with the highest stroke death rates were outside the 30-minute drive-time areas (Figure 2). Twenty-eight PCNASR and GWTG–Stroke hospitals that are not JCPSCs were in or near these areas of high stroke death rates (Figure 3). Most of the tri-state population was within 30-minute (75%) and 60-minute (95%) drive times to either a JCPSC, PCNASR, or GWTG–Stroke hospital (Table). The sociodemographic disparities in the population distribution within 30- and 60-minute drive times were maintained after the inclusion of PCNASR and GWTG–Stroke hospitals (Table).

## Discussion

We found substantial geographic and sociodemographic disparities in drive times to JCPSCs in the tri-state region of North Carolina, South Carolina, and Georgia. Overall, approximately 55% of the population in the tri-state region lived within a 30-minute drive time to a JCPSC and 77% lived within a 60-minute drive time. For 30-minute and 60-minute drive times, people in younger age groups, with higher education levels, living in urban areas, and not living in poverty had more timely access to a JCPSC. When hospitals currently engaged in stroke care quality improvement initiatives (ie, the PCNASR and GWTG–Stroke hospitals) were included in the analysis, we observed substantial increases in percentage of the population within 30-minute and 60-minute drive times, suggesting that these hospitals are well located to serve populations that do not have timely access to JCSPCs.

These results highlight opportunities to strengthen stroke systems of care by including hospitals that may not meet the criteria for designation as a JCPSC but have the potential to provide acute stroke treatment services. The 2 main avenues for expanding the role of these hospitals are 1) establishment of an additional set of evidence-based criteria for acute stroke treatment center designation that complements but does not replace the JCPSC criteria (not unlike the multilevel trauma center designation) and 2) enhancement of telestroke networks.

An additional set of evidence-based stroke center criteria to complement the existing JCPSC criteria would enhance stroke systems of care by recognizing the vital role that smaller hospitals fill in treating stroke patients, especially in rural areas such as those in the tri-state region of North Carolina, South Carolina, and Georgia. Healthcare Facilities Accreditation Program is 1 example of an additional stroke center certification program that has certified 13 primary stroke centers nationwide (www.hfap.org/AccreditationPrograms/acute.aspx).

Several states have already begun to develop alternative stroke center designation programs. However, many of these programs lack the infrastructure to verify (with onsite inspection) whether hospitals or facilities can appropriately be classified as stroke centers. Hospitals relying on remote survey techniques to self-report their stroke treatment capabilities may overestimate their stroke service capabilities (20). An additional set of evidence-based criteria established by stroke experts that would be rigorously verified would provide regional, state, and local organizations an opportunity to continue their collaborative efforts of enhancing stroke systems of care by strategically identifying where stroke centers are most needed and what resources would be needed to enable existing hospitals to perform key functions for acute stroke care.

Telestroke refers to the application of telemedicine in stroke care, in which consultation is performed by a remotely located expert through the use of high-quality videoconferencing. This type of remote evaluation of stroke patients is increasingly being used and strengthens state and regional stroke systems of care (21,22). The American Heart Association recognizes the importance of telemedicine for improving stroke care, especially in rural areas where facilities are scarce and emergency medical services transport can be inadequate (12,23). Telestroke networks exist in North Carolina, South Carolina, and Georgia, and Georgia plans to expand the scope of its telestroke networks (24-26). The existing telestroke networks are unable to provide telestroke services to some of the high-risk areas identified in this study (ie, most of the coastal plains of North Carolina and South Carolina that are outside a 60-minute drive time to a JCPSC and have high rates of stroke death).

Two other studies have estimated the percentage of the population living within a 60-minute drive time to stroke centers, with results similar to those of this study. In Canada, 67% of the total population lived within a 60-minute drive time to a hospital capable of administering tPA (27). In the United States, a report by the Northwest Regional Stroke Network found that 69% of its population was within a 60-minute drive time to a JCPSC (28). These studies suggest that although the rural areas of the tri-state region are neither as vast nor as remote as rural areas in the Northwest and Canada, a liberal 60-minute travel window around existing JCPSCs still leaves a similar percentage of the population (more than 30%) without timely access to JCPSCs. Other studies of drive times to acute stroke care have used different time frames or travel distances. In Georgia, 69% of the population was determined to be living within 20 miles of a stroke-ready hospital and in North Carolina, 21% of stroke deaths were among patients who lived within a 20-minute drive to a JCPSC (29,30).

The use of Network Analyst provides a more accurate representation of drive times than the more commonly used uniform distance buffers. However, drive-time analyses are limited because neither approach is able to account for changes in the elevation of the terrain or faster driving speed by emergency medical services. Consequently, the drive times in mountainous areas may be underestimated and may be overestimated in settings where emergency medical services are readily available and traffic patterns allow higher speeds. Another limitation of our study is the use of death certificate data for calculating stroke death rates. Geographic variations in reporting practices could introduce bias into the geographic patterns of stroke death rates. However, past studies have not documented a systematic geographic bias in the accuracy of reporting stroke deaths (31).

In summary, many geographic areas in the tri-state region of North Carolina, South Carolina, and Georgia, an area of the country with a historically high stroke death rate, lack timely access to JCPSCs. The results of this study highlight the need for alternative strategies to expand provision of quality acute stroke care in the tri-state region, particularly to underserved populations. Application of the GIS technique used in this study visually portrays the population effect of selected scenarios for improving access to timely acute stroke care.

## Figures and Tables

**Table:** Population Within Specified Drive Times to Hospitals With a Primary Stroke Center or a Stroke Care Quality Improvement Initiative, by Demographic Characteristics, North Carolina, South Carolina, and Georgia^a^

Characteristic	N^b^	% Within Drive Time to JCPSCs Only^c^		**% Within Drive Time to JCPSC, PCNASR, or GWTG**–**Stroke Hospitals^d^**	

		30 min	60 min	30 min	60 min
Total	20,247,778	54.8	77.3	75.3	94.7
**Age, y**					
<40	11,937,561	56.5	78.3	76.9	95.1
40-49	3,028,225	55.6	77.9	75.9	94.8
50-64	3,042,336	51.9	75.7	72.7	93.8
≥65	2,239,656	48.9	73.7	70.0	93.1
**Sex**					
Female	10,329,041	54.9	77.3	75.3	94.6
Male	9,918,737	54.8	77.3	75.3	94.7
**Race/ethnicity**					
Non-Hispanic black	5,272,303	53.9	73.8	75.4	94.8
Hispanic	909,266	68.3	85.3	83.5	96.8
Non-Hispanic white	13,827,497	54.3	78.1	74.6	94.4
**Education (age ≥25 y)**					
<High school	2,880,352	46.0	73.9	67.7	94.2
High school graduate	3,767,038	48.8	74.9	70.8	94.1
>High school	6,417,579	62.1	80.3	80.6	94.8
**Region^e^**					
Urban	13,140,663	70.4	84.0	87.7	96.1
Rural	7,107,115	26.2	65.0	52.4	91.9
**Income^f^**					
<Poverty level	2,540,329	47.3	71.0	69.1	93.4
≥Poverty level	17,107,997	56.2	78.5	76.3	94.8

Abbreviations: JCPSC, Joint Commission–Certified Primary Stroke Center; PCNASR, Paul Coverdell National Acute Stroke Registry; GWTG–Stroke, Get With the Guidelines–Stroke program.

a Source: US Census 2000 Summary File 1 and Summary File 3 (16,17).

b Values for each category may not add to total because of missing data.

c All characteristics are significant at *P* < .001 except sex. Calculated by using χ^2^ test.

d All characteristics are significant at *P* < .001 except sex for 30-minute drive times. Calculated by using χ^2^ test.

e Urban defined as urbanized areas (densely settled territories with ≥50,000 people) and urban clusters (densely settled territories with >2,500 people but <50,000 people). Rural defined as all other areas.

f 1999 poverty level derived from 2000 US Census (17).
